# Niflumic Acid Reverses Airway Mucus Excess and Improves Survival in the Rat Model of Steroid-Induced *Pneumocystis* Pneumonia

**DOI:** 10.3389/fmicb.2019.01522

**Published:** 2019-07-05

**Authors:** Francisco J. Pérez, Pablo A. Iturra, Carolina A. Ponce, Fabien Magne, Víctor Garcia-Angulo, Sergio L. Vargas

**Affiliations:** Programa de Microbiología y Micología, Instituto de Ciencias Biomédicas (ICBM), Facultad de Medicina, Universidad de Chile, Santiago, Chile

**Keywords:** *Pneumocystis* pneumonia, steroid-resistant-mucus, animal model, mucus, Innate Immunity, CLCA1, MUC5AC, niflumic acid

## Abstract

Although the role of adaptive immunity in fighting *Pneumocystis* infection is well known, the role of the innate, airway epithelium, responses remains largely unexplored. The concerted interaction of innate and adaptive responses is essential to successfully eradicate infection. Increased expression of goblet-cell-derived CLCA1 protein plus excess mucus in infant autopsy lungs and in murine models of primary *Pneumocystis* infection alert of innate immune system immunopathology associated to *Pneumocystis* infection. Nonetheless, whether blocking mucus-associated innate immune pathways decreases *Pneumocystis*-related immunopathology is unknown. Furthermore, current treatment of *Pneumocystis* pneumonia (PcP) relying on anti-*Pneumocystis* drugs plus steroids is not ideal because removes cellular immune responses against the fungal pathogen. In this study, we used the steroid-induced rat model of PcP to evaluate inflammation and mucus progression, and tested the effect of niflumic acid (NFA), a fenamate-type drug with potent CLCA1 blocker activity, in decreasing *Pneumocystis*-associated immunopathology. In this model, animals acquire *Pneumocystis* spontaneously and pneumonia develops owing to the steroids-induced immunodeficiency. Steroids led to decreased animal weight evidencing severe immunosuppression and to significant *Pneumocystis*-associated pulmonary edema as evidenced by wet-to-dry lung ratios that doubled those of uninfected animals. Inflammatory cuffing infiltrates were noticed first around lung blood vessels followed by bronchi, and both increased progressively. Similarly, airway epithelial and lumen mucus progressively increased. This occurred in parallel to increasing levels of MUC5AC and mCLCA3, the murine homolog of hCLCA1. Administration of NFA caused a significant decrease in total mucus, MUC5AC and mCLCA3 and also, in *Pneumocystis*-associated inflammation. Most relevant, NFA treatment improved survival at 8 weeks of steroids. Results suggest an important role of innate immune responses in immunopathology of steroid-induced PcP. They warrant evaluation of CLCA1 blockers as adjunctive therapy in this condition and describe a simple model to evaluate therapeutic interventions for steroid resistant mucus, a common condition in patients with chronic lung disease like asthma, chronic obstructive pulmonary disease (COPD) and cystic fibrosis.

## Introduction

Progression of a mild fungal infection by *Pneumocystis* to the life-threatening *Pneumocystis* pneumonia (PcP) occurs in immunocompromised hosts largely because T-cell defects halt the coordinated action of the innate and adaptive immune systems required to clear infections (Iwasaki and Medzhitov, 2015). Research attention has mainly focused on the adaptive response. However, airway mucus is an essential component of the innate immune defense mechanisms of the lung and finely regulated mucus levels are critical for effective airway mucociliary clearance and lung health (Fahy and Dickey, 2010; Perez B.F. et al., 2014; Ha and Rogers, 2016).

Recent reports describe increased production of mucus and of specific MUC5AC and MUC5B mucus proteins during *Pneumocystis* primary infection in infants (Vargas et al., 2013; Perez F. J. et al., 2014; Rojas et al., 2019) and in immunocompetent rodent models (Meissner et al., 2005; Hernandez-Novoa et al., 2008; Vargas et al., 2013; Eddens et al., 2016; Rojas et al., 2019). Importantly, mucus excess as a pathologic feature in PcP remains unexplored. “Hydropically swollen muco-proteins” were histochemically identified by H. S. Baar in 1955 within the amorphous foamy material or acellular eosinophilic exudate characteristic of PcP thus emphasizing the increased fluid accumulation aspect of PcP related pathology (Baar, 1955). Characterization of the role of mucus in PcP is therefore a priority as excess airway mucus indicates inflammation, may limit airflow, impair mucociliary clearance and favor mucostasis, airway collapse and the development of mucus plugging which may therefore, contribute to the respiratory failure in PcP (Fahy and Dickey, 2010; Ha and Rogers, 2016; Perez B.F. et al., 2014; Ma et al., 2018).

Unfortunately, there is not an effective treatment for mucus hypersecretion. The options are few (Ha and Rogers, 2016), and they have limited efficacy in part explained by the multiple inflammatory pathways, inflammatory mediators and cytokines that drive mucus production (Hauber and Zabel, 2008; Ha and Rogers, 2016). Even corticosteroids, the best anti-inflammatory drug available, have low efficacy in PcP (Wieruszewski et al., 2018) and are unable to suppress goblet cell hyperplasia (Kibe et al., 2003; Nakano et al., 2006), emphasizing the need to investigate the mechanistic insights of excess mucus production associated to this fungal infection. Relevant to this research, steroids are strong inducers of PcP in cancer and other diseases in a dose dependent manner (Park et al., 2018). The capacity of steroids to induce PcP has been utilized for decades to model PcP in animals for *Pneumocystis* research. PcP develops in nearly 100% of the animals after administration of glucocorticoids during a period of 8 to 10 weeks (Hughes et al., 1994). The more accepted explanation for this effect is that systemic use of corticosteroids leads to profound cell-mediated immunosuppression encompassing apoptosis of immune cells including CD4+, CD8+, and other T cell lymphocyte subsets (Walzer et al., 1984; Barnes, 2016). This blunting of the adaptive immune responses halts the clearance of the infection (Iwasaki and Medzhitov, 2015) and therefore, allows a growing fungal burden of *Pneumocystis* in the lungs (Screpanti et al., 1989; Adcock and Mumby, 2017; Hu et al., 2017; Rong et al., 2018) that keeps stimulating the airway epithelium (Screpanti et al., 1989; Swain et al., 2012; Eddens et al., 2016; Adcock and Mumby, 2017; Iturra et al., 2018). Collectively, available data suggest that *Pneumocystis* infection overcomes the anti-inflammatory effects of corticosteroids and induces a steroid-resistant mucus phenotype in this model.

Host recognition of *Pneumocystis* and triggering of immune responses is an area of intense research (Hoving and Kolls, 2017; Hoving, 2018; Hauser, 2019). The cyst (ascus) and trofozoite (nuclei) forms exhibit different antigens and both display differing strategies to evade host recognition (Hoving and Kolls, 2017; Hoving, 2018; Hauser, 2019). It is well described that β-glucans present in the thick *Pneumocystis* cyst wall are recognized by host pattern-recognition receptors such as Dectin-1 and Mincle located in macrophages and by HSPA5 in the airway epithelium thereby activating airway innate immune responses (Krajicek et al., 2009; Ricks et al., 2013; Hoving and Kolls, 2017; Hoving, 2018; Kottom et al., 2018; Hauser, 2019). *Pneumocystis*-mediated overexpression of the goblet-cell-derived calcium-activated chloride channel (CLCA) regulator CLCA1, also known as Gob5 or mCLCA3 (the murine homolog), was first suggested by Kovacs et al. using microarray technology (Hernandez-Novoa et al., 2008), and more recently confirmed by us and others (Swain et al., 2012; Perez F. J. et al., 2014; Iturra et al., 2018). CLCA1 is a secreted signaling protein that regulates airway target cells in healthy and disease conditions with a role in the development of mucous metaplasia and increased airway mucus production (Patel et al., 2009; Sala-Rabanal et al., 2015). Consensus STAT6-binding sites have been identified in the mClca3 and human Clca1 gene regulatory regions suggesting that it directly mediates responsiveness to IL-13 stimulation (Patel et al., 2009). *Pneumocystis* elicits a STAT6 innate immune response that can result in airway hyperresponsiveness (Swain et al., 2012). The emerging evidence of excess mucus and goblet cell metaplasia associated to CLCA1 pathway activation by *Pneumocystis* with upregulation of MUC5AC and MUC5B mucin expression suggests that this fungus induces strong stimuli of mucus related host airway responses whose role in immunopathology warrants characterization (Patel et al., 2009; Swain et al., 2012; Perez F. J. et al., 2014; Iturra et al., 2018).

We hypothesized that the persistent stimulation of CLCA1 mediated immune responses in the airway epithelium by *Pneumocystis* leads to mucus related immunopathology, and selected the classic steroid-induced rat model of PcP to characterize the histological progression of mucus and to further do a case control experiment blocking CLCA1 to evaluate the role of this protein in this immunopathology. We selected this rat model because resembles the circulation of *Pneumocystis* in the human community where the infection is unnoticeably acquired in a non-closed environment, and furthermore, because the progression of *Pneumocystis* infection to PcP is highly host-dependent, determined by host factors, like apoptosis of T-cell CD4 counts, that in this model are induced by administration of high-dose steroids (Walzer et al., 1984; Sukura et al., 1995). More importantly, steroids represent the most relevant risk factor for development of PcP in non-HIV-infected population (Park et al., 2018; Wieruszewski et al., 2018). In addition, we selected niflumic acid (NFA), a non-steroidal anti-inflammatory drug, because NFA has a potent, although not completely specific, blocker activity over the CLCA family of proteins (Famaey, 1997; Parai and Tabrizchi, 2002; Zhou et al., 2002; Ledoux et al., 2005; Nakano et al., 2006; Fukuyama et al., 2009). Of relevance, it is well documented that NFA inhibits goblet cell hyperplasia, mucus overproduction and airway hyperresponsiveness in mice as well as in human bronchial epithelial cells, through the reduction of hCLCA1 and MUC5AC expression (Hauber et al., 2005; Nakano et al., 2006; Yasuo et al., 2006; Hegab et al., 2007; Kim et al., 2007; Fukuyama et al., 2009). Documenting an effect of NFA in decreasing steroid resistant *Pneumocystis*-related immunopathology would lead to evaluate anti-CLCA1 related drugs as novel adjuvant treatments for PcP.

## Materials and Methods

### Ethics Statement

Ethical approval was obtained from the Institutional Animal Welfare Ethics Committee of the University of Chile School of Medicine (Santiago, Chile) under protocol number CBA0634. Animal experiments were conducted in accordance to the Animal Protection Law of Chile (Law 20.380) and following international directions of the Guide for the Care and Use of Laboratory Animals (Eighth Edition, National Academies Press, Washington, DC).

### Animal Model of Steroid-Induced *Pneumocystis* Pneumonia

**FIGURE 1:**
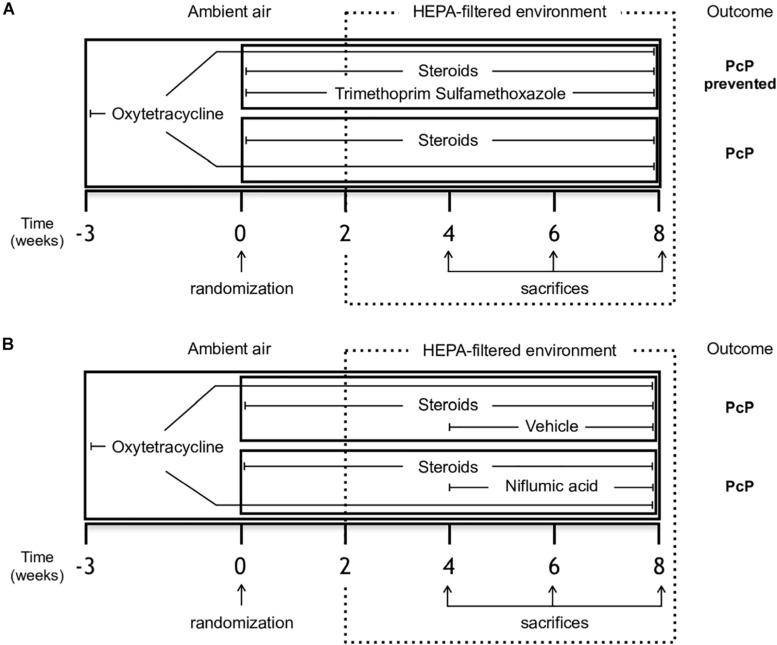
Study design. Female Sprague-Dawley rats from a single colony were treated with oxytetracycline starting 3 weeks before immunosuppressive regimen and kept throughout the experiment in both groups to prevent bacterial infections. **(A)** Experiment 1 - Rats were randomized to receive either steroids (*Pneumocystis*-infected group) or steroids plus anti-*Pneumocystis* prophylaxis consisting of trimethoprim/sulfamethoxazole (controls) for 8 weeks. **(B)** Experiment 2 - Rats receiving steroids and oxytetracycline were randomized at week 4 to continue with steroids plus NFA treatment (6 mg/Kg/day) or to steroids plus the vehicle solution without NFA (control group) for additional 4 weeks. Eight rats per group were sacrificed on weeks 4, 6, and 8 of immunosuppression. At each time point, four rats per group underwent vascular perfusion of lungs *in situ* using buffered formalin to preserve lung architecture for histological analysis. Lungs from the other four rats per group were fresh-extracted for use in molecular determinations. HEPA: High-Efficiency Particulate Air; NFA: Niflumic acid (6 mg/Kg/day); Pc (−): uninfected control group; Pc (+): *Pneumocystis*- infected group.

Sprague Dawley juvenile female rats (180–200 g body weight) from a single colony were used in each experiment. They were housed in a standard animal room to let them acquire *Pneumocystis* from the air and given oxytetracycline (0.4 mg/mL) in the drinking water starting 3 weeks prior to the start of immunosuppression and maintained throughout the experiment to eliminate eventual respiratory bacterial pathogens, specially *Mycoplasma pulmonis* (Banerjee et al., 1987). PcP was induced using high dose betamethasone (3 mg/L). This experimental scheme is highly effective in inducing PcP and cysts can be detected after 2 weeks of immunosuppression (Hughes et al., 1974). Two animals per experimental group were sacrificed at the end of week two to confirm *Pneumocystis* in lungs by microscopy using Grocott-Gomori methenamine silver stain before the animals were moved a high-efficiency particulate-filtered air environment (One Cage 2100, Lab Products Inc.) to prevent acquisition of new infections. Corticosteroids were given for a total of 8 weeks. A control group of rats given anti-*Pneumocystis* prophylaxis with trimethoprim (50 mg/Kg) sulfamethoxazole (250 mg/Kg) (TMP-SMZ) (Hughes et al., 1974) *ad libitum* in the drinking water was used for comparison to characterize the progression of mucus, inflammation and lung edema (Figure 1A). The individual weight of the animals was recorded weekly.

### Niflumic Acid Administration

The effect of NFA was evaluated in animals with steroid-induced PcP. Animals were randomized to receive NFA or vehicle (placebo). The NFA dose used was 6 mg/Kg/day, 7 days/week, starting at week 4 of steroid administration. NFA (Sigma-Aldrich) was dissolved daily in 0.4 M NaHCO_3_ in 5% glucose, adjusted to pH 7.5, passed through 0.22 μm filters and administered via the intraperitoneal (IP) route. Control animals received equivalent volumes of filtered 0.4 M NaHCO3 in 5% glucose pH 7.5 alone as vehicle/placebo (Figure 1B). The dose of NFA was chosen based on previous reports of *in vivo* experiments (Parai and Tabrizchi, 2002; Nakano et al., 2006; Hegab et al., 2007) and confirmed after in-house safety evaluations. Liver toxicity of 3 and 12 mg/kg/day dose was evaluated by measuring liver function tests in serum samples of 5 healthy rats per dose IP and of control rats receiving vehicle alone, daily, for 28 days prior to the start of experiments (Supplementary Figure S1).

### Lung Samples

Eight rats and their respective controls given TMP-SMZ were subject to the experimental scheme depicted in Figure 1A and let to develop PcP to evaluate body weight progression during steroid-induced immunosuppression, pulmonary edema, and survival at 8 weeks. Rats were sacrificed by exsanguination under deep anesthesia with ketamine (100 mg/kg) and xylazine (10 mg/kg), their lungs were extracted immediately, weighed, and put in an oven at 85°C during 24 h to determine the wet-to dry lung weight ratio as a measure of PcP-associated pulmonary edema. In subsequent experiments eight animals per group were sacrificed at 4, 6, and 8 weeks of immunosuppression under deep anesthesia as described above. Half of them were exsanguinated, their lungs removed, and their upper right lung lobes immediately separated, cut and stored at −20°C until protein extraction. Lungs in the other half were fixed using *in situ* vascular-perfusion as previously reported (Iturra et al., 2018). Briefly, 3.7% PBS-buffered formalin (pH 7.2) was perfused via the inferior cava vein at a pressure of 25 cm H_2_O. Perfused lungs were maintained inside the thorax for 12 h at room temperature and then were extracted and immersed in buffered formalin for additional 12 h. The upper right lung lobe was dissected, and paraffin-embedded for histology sections.

### Pneumocystis Diagnosis, Histologic, and Morphometric Assessments

Longitudinal 5 μm-thick lung tissue sections were observed using an OLYMPUS BX60 microscope connected to a QImaging MicroPublisher 3.3 RTV camera (QImaging). Morphometry assessments were performed by observers that were unaware of the experimental group using the Image-Pro Plus software version 5.1 (Media Cybernetics, Inc.). *Pneumocystis* was examined in lung imprints using Grocott-Gomori methenamine silver stain and pneumonia confirmed by Hematoxylin and Eosin (H&E) stain (Hughes et al., 1974). Peribronchial and perivascular inflammation was evaluated in lung sections stained with H&E stain measuring cellular cuffs around <300 μm bronchioles and its associated blood vessels using a modified semiquantitative scoring system as described (Iturra et al., 2018). Briefly, 0 indicates no surrounding cuffs seen; 1: cuffs in <25% of bronchioles or vessels; 2: cuffs in 25% to 50% of bronchioles or vessels; and 3: cuffs in >50% of bronchioles or vessels. Mucus was assessed in lung sections stained with Alcian blue/periodic acid-Schiff (AB/PAS) stain and evaluated for the presence of mucin glycoconjugates in the epithelium and in the lumen. The AB/PAS-positive epithelium area and the percentage of lumen area occupied by mucus were quantified separately. Morphometry measurements were done in five randomly selected <300 μm bronchioles per animal in all four rats per group/time point (using a random table). *Pneumocystis* DNA was studied at the end of the experiment 2 (week 8) in all animals that received steroids or steroids plus NFA, using DNA amplification as described (Vargas et al., 1995).

### Western Blotting

A 300 mg aliquot of lung tissue were disrupted with Tissue Tearor (BioSpec Products Inc.) in chilled modified RIPA buffer (50 mM Tris–HCl pH 7.4; 150 mM NaCl; 1 mM EDTA; 1% NP-40; 0.5% sodium deoxicolate; 1 mM PMSF; 1 μg/ml of each Aprotinin, Leupeptin, and Pepstatin). Total protein was quantified in supernatants using the Bradford assay (BIO-RAD). 30 μg protein samples were subjected to SDS-PAGE with 4% stacking and 8% resolving Tris-Glycine gels. Proteins were transferred to polyvinylidene difluoride membranes and blocked with 5% low-fat milk. Mouse anti-MUC5AC IgG antibody (1:500, 45M1, Santa Cruz Biotechnology) and Goat anti-mouse IgG-HRP antibody (1:2000, Santa Cruz Biotechnology) were used to detect MUC5AC. Rabbit anti-CLCA3 IgG antibody (1:200, Santa Cruz Biotechnology) and Chicken anti-rabbit IgG-HRP antibody (1:2000, Santa Cruz Biotechnology) were used to detect mCLCA3. Membranes were stripped, blocked and reprobed for Actin detection using a Goat anti-Actin IgG (1:500, Santa Cruz Biotechnology) and a Donkey anti-goat IgG-HRP (1:2000, Santa Cruz Biotechnology) antibodies. Enhanced chemiluminescence reagent was used for membrane development (Pierce ECL) with X-ray films (CL-XPosure, Thermo Fisher Scientific). Films were analyzed using ImageJ software (NIH, United States).

### Data Analyses and Statistics

Data was expressed as mean ± SD. Groups were compared using one-way ANOVA with Tukey *post hoc* test. When two factors were evaluated a two-way ANOVA with Bonferroni *post hoc* test was performed. Survival analysis was performed using the Kaplan-Meier estimator and survival distributions were compared using the Mantel-Cox test. In all data analysis values of *p* < 0.05 were considered significant. Statistical analysis was performed using Prism 5.0 software (GraphPad Software, Inc.).

## Results

### Steroid Induced PcP Model

#### Effect of Steroids in Weight, *Pneumocystis*, Lung Edema, and Survival

**FIGURE 2:**
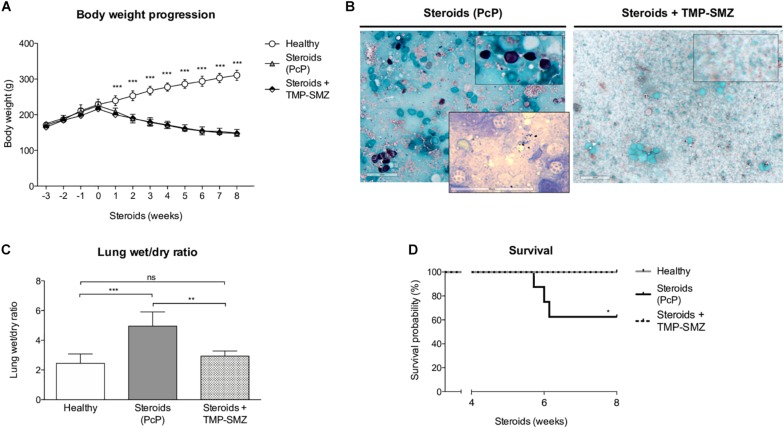
Effect of steroids on body weight, *Pneumocystis* infection, pulmonary edema, and PcP-associated survival. **(A)** Progression of body weight in animals receiving steroids with and without anti-*Pneumocystis* prophylaxis and in healthy controls. **(B)** Grocott-Gomori silver methenamine stain of *Pneumocystis* cysts in lung imprints of rats after 8 weeks of receiving steroids alone versus steroids plus anti-*Pneumocystis* prophylaxis with trimethoprim sulfamethoxazole. Insert documenting nuclei forms of *Pneumocystis* using Giemsa stain in rats without prophylaxis. Scale bar = 50 μm. **(C)** Pulmonary fluid accumulation determined by the wet / dry lung weight ratios at 8 weeks of steroids administration. **(D)** Kaplan–Meier survival plots at the eighth week of immunosuppression of rats healthy rats, with PcP, and rats receiving PcP prophylaxis. PcP: *Pneumocystis* pneumonia; TMP-SMZ: Trimethoprim Sulfamethoxazole. **(A,C)** Data are expressed as mean ± SD; **(A)** *n* = 8; ANOVA test: ^∗∗∗^*P* < 0.001; **(C)** *n* = 5; ANOVA test: ^ns^no significant; ^∗∗^ *P* < 0.01; ^∗∗∗^*P* < 0.001. **(D)** *n* = 8; Mantel-Cox test: ^*^P < 0.05. Dataset for this figure available in Supplementary Data Sheet S1.

We evaluated body weight changes, lung fluid accumulation and survival in the steroid-induced PcP model rats as per “A” experimental scheme (Figure 1). Animals receiving steroids steadily lost body weight regardless of anti-*Pneumocystis* prophylaxis while control rats without steroids progressively gained body weight (Figure 2A). This group of rats was sacrificed at the eighth week of immunosuppression and *Pneumocystis* cysts were detected in lung imprints of all animals receiving steroids and not detected in those receiving TMP-SMZ or in the healthy controls (Figure 2B). Increased weight of the wet portion of the lung was detected in the *Pneumocystis*-infected animals. The wet-to-dry lung ratio, a measure of lung edema, increased in the *Pneumocystis* infected animals with respect to healthy controls and to rats receiving anti-*Pneumocystis* prophylaxis (Figure 2C). No differences in the dry lung weight were detected across all groups (data not shown). Survival of rats with PcP rounded 60% at week eighth of steroid administration and TMP-SMZ was 100% efficient in preventing *Pneumocystis* and associated mortality (Figure 2D).

### Steroid-Induced PcP Promotes Perivascular and Peribronchial “Cuffing” Inflammation and Excess in Total Mucus With Increased MUC5AC and mCLCA3 Mucus Markers

#### *Pneumocystis* Induces Progressive Increase in Peribronchial and Perivascular Inflammatory Cuffs

**FIGURE 3:**
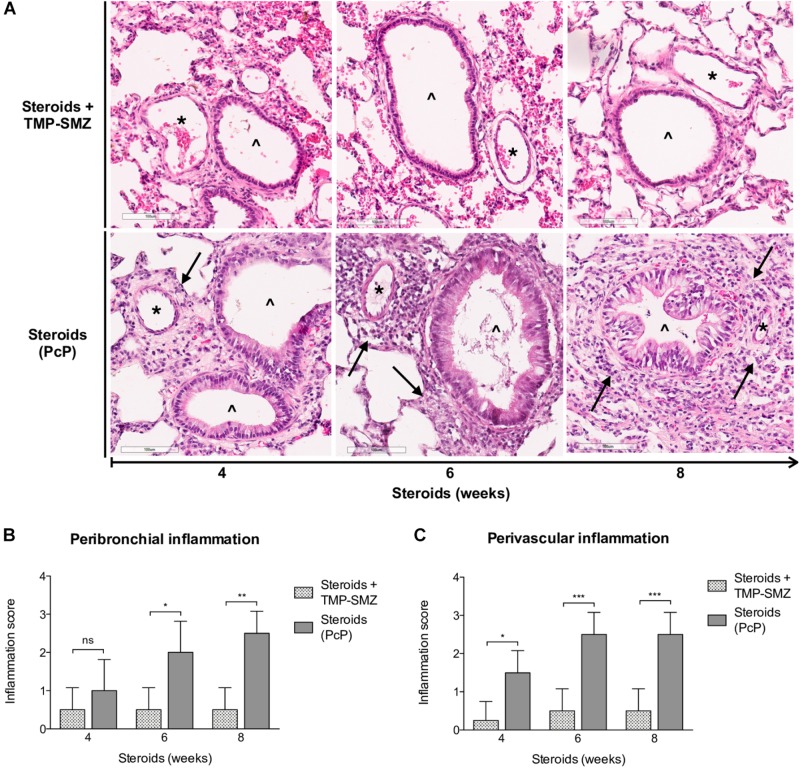
Sequential inflammatory changes in rats on steroids receiving and not-receiving anti-*Pneumocystis* prophylaxis. **(A)** Representative microscopic images of H&E-stained lung sections at 4, 6, and 8 weeks of immunosuppression. Blood vessels (^*^); bronchioles (^∧^); PcP: *Pneumocystis* pneumonia; TMP-SMZ: Trimethoprim Sulfamethoxazole. H&E stain, Scale bar = 100 μm. **(B,C)** Proportion of bronchioles and blood vessels surrounded by inflammatory cuffs infiltrates classified using a semiquantitative score (see Section “Materials and Methods”). Data are expressed as mean ± SD; *n* = 4; ANOVA test: ^ns^no significant; ^*^P < 0.05; ^∗∗^*P* < 0.01; ^∗∗∗^*P* < 0.001. Dataset for this figure available in Supplementary Data Sheet S1.

Inflammatory response as assessed in H&E-stained lung sections at 4, 6, and 8 weeks of steroid administration documented a progressive increase in *Pneumocystis*-associated inflammatory cuffs surrounding blood vessels and bronchioles (Figure 3A arrows). Semiquantitative score measurements showed that cellular cuff infiltrates appeared significantly earlier around blood vessels than around bronchioles (4 versus 6 weeks of immunosuppression). Control animals receiving TMP-SMZ prophylaxis did not develop significant inflammation (Figures 3B,C).

#### *Pneumocystis* Induces Progressive Increase of Total Mucus Production

**FIGURE 4:**
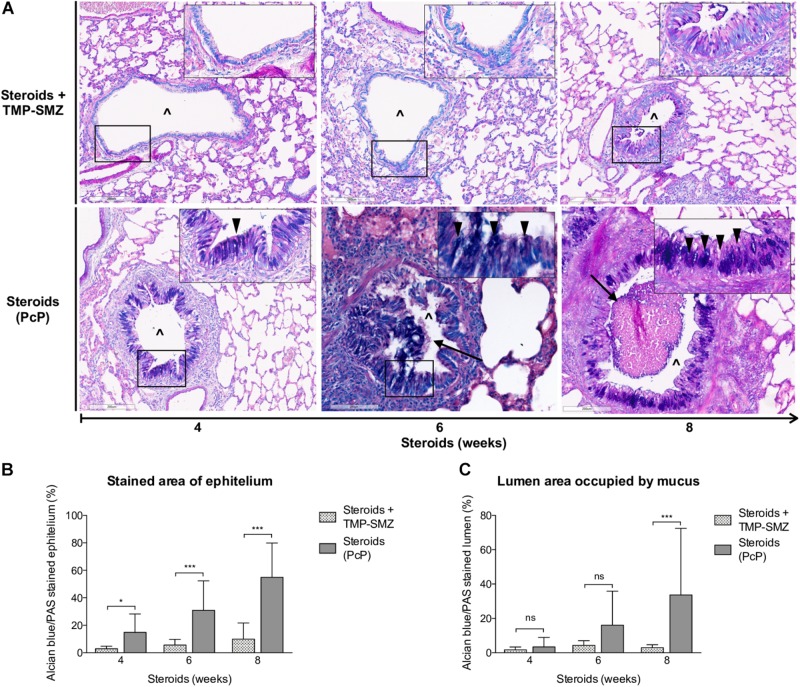
Sequential changes in mucus in rats on steroids receiving and not-receiving anti-*Pneumocystis* prophylaxis. **(A)** Representative microscopic images of AB/PAS-stained lung sections at 4, 6, and 8 weeks of immunosuppression. Intracellular mucin granules (arrowheads); Mucus plugs (arrows); PcP: *Pneumocystis* pneumonia; TMP-SMZ: Trimethoprim Sulfamethoxazole; (^∧^): Bronchiolar lumen. AB/PAS stain, Scale bar = 200 μm. **(B,C)** Percent of AB/PAS stained epithelium **(B)** and lumen **(C)**. Data are expressed as mean ± SD; *n* = 4; ANOVA test: ^ns^no significant; ^*^P < 0.05; ^∗∗∗^*P* < 0.001. Dataset for this figure available in Supplementary Data Sheet S1.

Progressive overproduction of mucus associated to *Pneumocystis* was documented by histology morphometry quantification of AB/PAS-stained area at 4, 6, and 8 weeks in rat lung sections of steroid-receiving rats (Figures 4A,B). Increase in intracellular mucin granules was also detected in the respiratory epithelium (Figure 4A, arrowheads). AB/PAS stained cells were absent to rare in control animals receiving TMP-SMZ at every measured time point (Figures 4A,B). Mucus plugs occupying the airway lumen increased in association to *Pneumocystis* with significant differences at 8 weeks of immunosuppression (Figures 4A arrows, 4C).

#### *Pneumocystis* Induces Progressive Increase in MUC5AC and mCLCA3 Protein Expression

**FIGURE 5:**
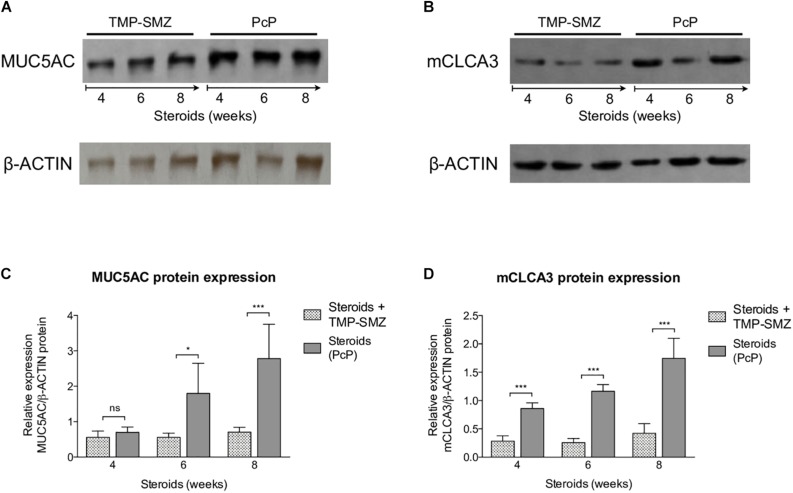
Sequential changes in MUC5AC and mCLCA3 protein levels in rats on steroids receiving and not-receiving anti-*Pneumocystis* prophylaxis (PcP). **(A,B)** Representative images of Western Blot analysis of MUC5AC **(A)** and mCLCA3 **(B)** protein expression levels in fresh lung tissue at 4, 6, and 8 weeks of immunosuppression. **(C,D)** Relative densitometric quantitation of MUC5AC **(C)** and mCLCA3 **(D)** expression. PcP: *Pneumocystis* pneumonia; TMP-SMZ: Trimethoprim Sulfamethoxazole. Data are expressed as mean ± SD; *n* = 4; ANOVA test: ^ns^no significant; ^*^P < 0.05; ^∗∗∗^*P* < 0.001. Dataset for this figure available in Supplementary Data Sheet S1.

A significant increase in MUC5AC (Figures 5A,C) and mCLCA3 (Figures 5B,D) expression was noticed at 6 and 4 weeks of steroid administration, respectively. Control animals showed no MUC5AC or mCLCA3 expression changes.

Taken together, the findings in rats with steroid-induced PcP indicate that *Pneumocystis* induces progressive inflammation, and mucus overproduction with increased expression of MUC5AC and mCLCA3.

### Niflumic Acid Is Well-Tolerated and Reverses Inflammation, Mucus Excess and Improves Survival in the Steroid-Induced PcP Model

#### Safety and Tolerance of Niflumic Acid

The safety of 3 and 12 mg/Kg/day NFA administered daily for 28 days intraperitoneally was evaluated in healthy rats prior to efficacy evaluations in PcP. Animals receiving NFA as well as the control rats receiving vehicle alone increased in body weight (Supplementary Figure S1A). Urea blood levels showed a dose-dependent increase in NFA-treated animals (Supplementary Figure S1B) and alkaline phosphatases increased in NFA 3 mg/Kg/day-treated animals compared to control rats (Supplementary Figure S1C). Liver (Total and conjugated bilirubin, AST, ALT, and GGT) and renal (creatinine) function tests were within reference ranges for Sprague-Dawley rats in all the animals. These findings indicate that intraperitoneal administration of NFA at these doses is safe and well tolerated by the animals.

#### NFA Administration Decreases Inflammation and Mucus Overproduction in Rats With Steroid Induced PcP

**FIGURE 6:**
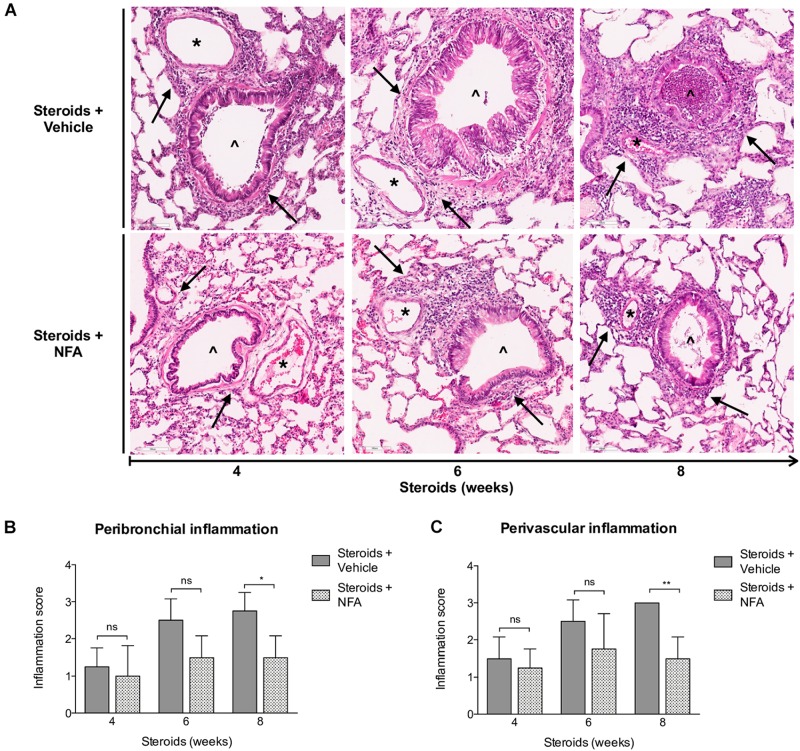
Effect of NFA in the inflammatory response during PcP. **(A)** Representative microscopic images of H&E-stained lung sections at 4, 6, and 8 weeks of immunosuppression. Cuff infiltrates (arrows); blood vessels (^*^); bronchioles (^∧^). NFA: Niflumic acid; Scale bar = 100 μm. **(B,C)** Proportion of bronchioles and blood vessels surrounded by inflammatory cuff infiltrates (see Section “Materials and Methods”). Data are expressed as mean ± SD; *n* = 4; ANOVA test: ^ns^no significant; ^*^P < 0.05; ^∗∗^*P* < 0.01. Dataset for this figure available in Supplementary Data Sheet S1.

**FIGURE 7:**
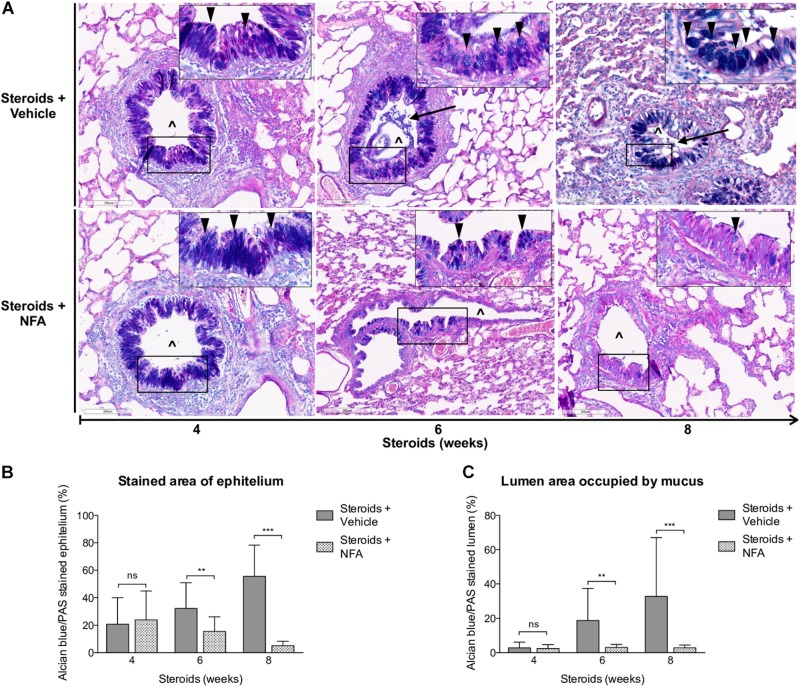
Effect of NFA in mucus production during PcP. **(A)** Representative microscopy images of AB/PAS-stained lung sections at 4, 6, and 8 weeks of immunosuppression. Intracellular mucin granules (arrowheads); mucus plugs (arrows); NFA: Niflumic acid; (^∧^): bronchiolar lumen; Scale bar = 200 μm. **(B,C)** Percent of AB/PAS stained epithelium **(B)** and lumen **(C)**. Data are expressed as mean ± SD; *n* = 4; ANOVA test: ^ns^no significant; ^∗∗^*P* < 0.01; ^∗∗∗^*P* < 0.001. Dataset for this figure available in Supplementary Data Sheet S1.

**FIGURE 8:**
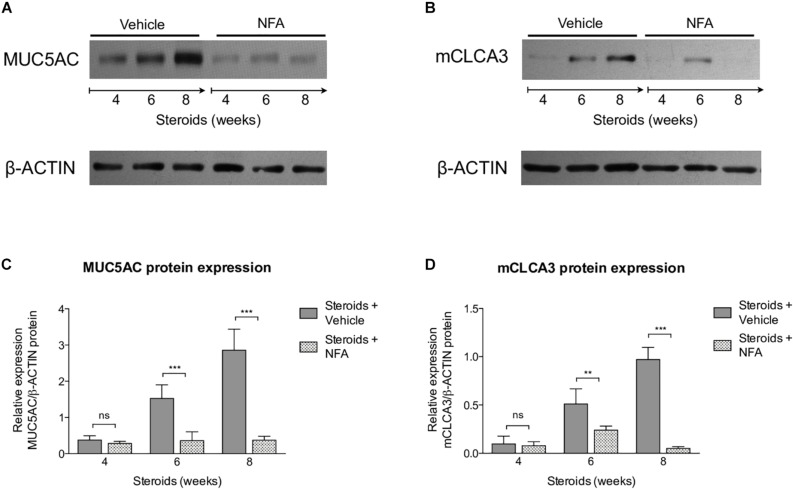
Effect of NFA in MUC5AC and mCLCA3 protein levels during PcP. **(A,B)** Representative images of Western Blot analysis of MUC5AC **(A)** and mCLCA3 **(B)** protein expression levels in fresh lung tissue of rats with or without NFA administration at 4, 6, and 8 weeks of immunosuppression. **(C,D)** Relative densitometric quantitation of MUC5AC **(C)** and mCLCA3 **(D)** expression. NFA: Niflumic acid. Data are expressed as mean ± SD; *n* = 4; ANOVA test: ^ns^no significant; ^∗∗^*P* < 0.01; ^∗∗∗^*P* < 0.001. Dataset for this figure available in Supplementary Data Sheet S1.

Next, steroids were administered to two groups of rats, with one group receiving NFA and the other receiving vehicle alone. *Pneumocystis* cysts were detected in lung imprints of all rats, regardless of whether they received NFA or vehicle (controls). In addition, *Pneumocystis* spp.-DNA was detected in all rats receiving steroids alone or steroids plus NFA (Supplementary Figure S2). Of relevance, treatment with NFA slowed the progression of cellular cuffing infiltrates around blood vessels (^*^) and bronchioles (**^∧^**) regardless of the presence of Pneumocystis (Figure 6A arrows) and this decrease in progression reached significance respect to vehicle at the eighth week of steroid administration, corresponding to 4 weeks of continuous NFA treatment (Figures 6B,C). Animals that receive vehicle alone showed progressive inflammation. Furthermore, NFA stopped and reverted mucus overproduction as evidenced by a significant decrease of AB/PAS-stained area (Figures 7A,B), a reduction of intracellular mucin granules in the epithelia (Figure 7A arrowheads) and a depletion of mucous plugs in airway lumen (Figures 7A arrows, 7C). The reduction in mucus production became significant after 2 weeks of NFA continuous administration (6 weeks of steroids) (Figures 7B,C). Rats that received placebo (steroids + vehicle) showed progressive mucus overproduction. Moreover, the continuous administration of NFA suppressed the increased expression of both MUC5AC (Figures 8A,C) and mCLCA3 (Figures 8B,D) starting from 2 weeks of NFA treatment.

#### Niflumic Acid Increases Survival in Animals With Steroid-Induced PcP

**FIGURE 9:**
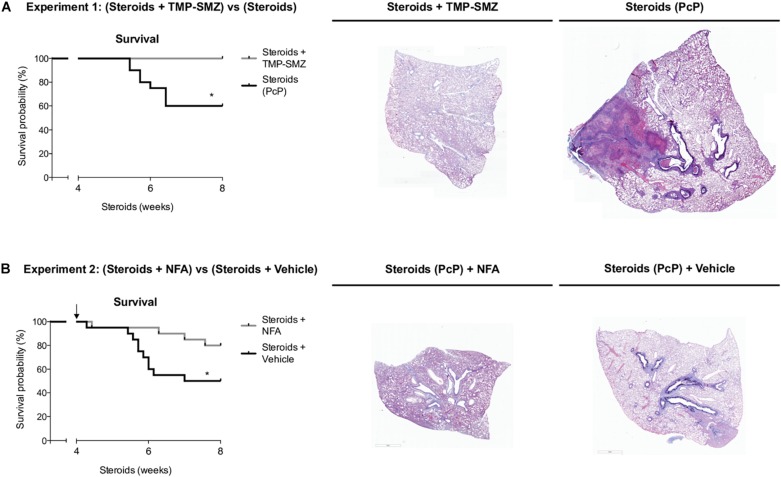
Niflumic acid treatment improves animal survival in PcP. Kaplan–Meier survival plots and representative lung sections stained with Alcian Blue/PAS at 8 weeks of steroid treatment for animals with steroid-induced PcP versus animals receiving steroids plus anti-*Pneumocystis* prophylaxis with TMP-SMZ in experiment 1 **(A)**, or for animals receiving NFA plus steroids alone versus steroids plus placebo (NFA vehicle) in experiment 2 **(B)**. PcP: *Pneumocystis* pneumonia; TMP-SMZ: Trimethoprim Sulfamethoxazole; NFA: Niflumic acid. *n* = 20; Mantel–Cox test: ^*^P < 0.05. Dataset for this figure available in Supplementary Data Sheet S1.

Survival plots were determined for animals that received steroids versus animals that received steroids + TMP-SMZ (Experiment 1 - Figure 1A), and for animals receiving steroids versus steroids + NFA (Experiment 2 - Figure 1B). Prevention of *Pneumocystis* conferred a significant survival improvement compared to animals that developed PcP (100% versus 60%) (Figure 9A). Animals developing steroid-induced PcP in the second experiment had a survival of 60%; similar to the animals developing steroid-induced PcP in the first experiment. However, animals receiving continuous treatment with NFA for 4 weeks (28 days) reached an 80% survival that was significantly improved respect to animals receiving steroids and placebo (vehicle) (Figure 9B). This survival improvement was lost if administration of NFA was intermittent (i.e., 5 days / week) and animals interrupting NFA approached their survival values to those of non-treated animals in 2 days, at first interruption of NFA treatment (Supplementary Figure S3). Increased lung volumes leading to greater slice sections areas were noticed in lungs of rats with PcP (Steroids or Steroids + vehicle) compared to rats that received prophylaxis with TMP-SMZ. This is consistent with increased lung edema in rats with PcP (Figure 2C). Also, rats with PcP receiving NFA treatment had smaller lungs than those with PcP receiving steroids + placebo (vehicle). Rats with PcP in experiment 1 **(A)** or with steroids plus placebo (**B**) developed mucus plugs (see also Figures 4, 7), and collapse of important areas of the lung was frequently observed among them.

## Discussion

In this work we show that the *Pneumocystis*-induced mucus response of the airway epithelium is highly activated during PcP and can be attenuated by treatment with Niflumic acid. This suggests a relevant role for the innate immune system in immunopathology of this fungal infection. The pathology features documented in this model consisted of steroid-resistant mucus excess with significantly increased protein expression of MUC5AC and mCLCA3. All these endpoints were reverted by the administration of niflumic acid, a specific and strong CLCA1 blocker, albeit not full blocker of mucus responses and with pleiotropic anti-inflammatory effects. Moreover, rats receiving this drug had increased survival suggesting the important participation of CLCA1-associated mechanisms in mediating *Pneumocystis*-associated immunopathology.

The first experiments aimed to document the progression of the disease in the rat model of steroid-induced PcP (Figures 2–5). Importantly, in addition to pulmonary edema they showed that mucus excess is a relevant disease feature during PcP by histologically documenting airway plugs and extensive shunting (Figure 9A). Differences in lung size are attributed to slight differences in rat size, magnitude of lung edema, or genetic differences in response to PcP Further results suggest that the mechanism for this pathogenic feature is mediated by CLCA1 which hints into a strong involvement of innate immune pathways that have been previously associated with *Pneumocystis* infection in immune competent human and animal hosts (Wang et al., 2005; Hernandez-Novoa et al., 2008; Swain et al., 2012; Vargas et al., 2013; Bello-Irizarry et al., 2014; Perez F. J. et al., 2014; Eddens et al., 2016; Iturra et al., 2018) and could help explain the low sensitivity to high dose steroids observed in PcP (Maeda et al., 2015; Wieruszewski et al., 2018).

*Pneumocystis* pneumonia induced perivascular inflammatory infiltrates, mucus overproduction and increased mCLCA3 protein levels were noticed after 4 weeks of steroid-induced immunosuppression; at the first sacrifice date. Increase in peribronchial infiltrates and in MUC5AC protein levels followed. This sequence of progression of inflammatory changes was similar to the sequence documented previously in immunocompetent rats with primary infection (Iturra et al., 2018) (*P* < 0.001), (Figures 3, 4).

Extrapolation of this model to pathogenesis of specific types of PcP needs to be cautious. The immunopathogenesis of PcP is incompletely understood and may vary in patients depending on their underlying immunosuppressive etiology (Eddens and Kolls, 2015; Kutty et al., 2016; Bhagwat et al., 2018). This work did not measure T-cell levels. However, T-cell blunting is a well-documented feature of steroid-induced PcP and is expected as a result of steroid administration in this model (Walzer et al., 1984; Sukura et al., 1995). Therefore, the pathology features and response to NFA therapy determined here may not necessarily extrapolate to other types of PcP. For example, PcP in patients with immune reconstitution inflammatory syndrome (IRIS) display high lymphocyte counts after antiretroviral-therapy-related T-cell recovery (Eddens and Kolls, 2015; Gopal et al., 2017; Aggarwal et al., 2018). A progressive increase in CLCA1 as in this model may more likely occur in the setting of the slowly developing PcP of untreated AIDS or in cancer patients receiving steroids, than in the context of recovering CD4+ T-cell counts able to trigger a strong cellular immune response, or of other underlying etiologies.

The mucus excess in this model developed despite the potent anti-inflammatory activity of high-dose steroids. The effect of steroids was overcome by the effect of *Pneumocystis* in stimulating mucus-related pathways indicating that mucus in this model is steroid resistant. This may be partially explained because CLCA1 activates MAP kinases (Alevy et al., 2012), and MAP kinases phosphorylate the glucocorticoid receptor thereby inhibiting steroid signaling (Rhen and Cidlowski, 2005). However, there is currently no complete explanation for steroid resistant mucus (Barnes, 2013). Several mechanisms have been identified which include familial glucocorticoid resistance, glucocorticoid receptor modifications, increased glucocorticoid receptor-β expression, increased proinflammatory transcription factors, and defective histone acetylation (Barnes, 2016). Importantly, corticosteroids have been shown to be unable to control IL-13-induced mucus (Liu et al., 2004; Kanoh et al., 2011) and are also incapable of suppressing IL-13-induced goblet cell hyperplasia in mice (Kibe et al., 2003), suggesting that CLCA1-associated mucus response was beyond the anti-inflammatory reach potential of steroids (Kibe et al., 2003; Nakano et al., 2006; Alevy et al., 2012) as occurred in this model. Corticosteroids are used as adjuvant therapy in severe HIV-related PcP because inflammation is a well-known pathology feature of PcP and they are the best anti-inflammatory drugs available (Krajicek et al., 2009; Wieruszewski et al., 2018). Paradoxically, corticosteroids offer no therapeutic benefit to patients with Non-HIV-related PCP in whom inflammation is characteristically worse (Wieruszewski et al., 2018). Results of this model raise the hypothesis that the lack of response to steroid treatment observed in these patients reflects that a relevant proportion of their immunopathology is steroid-resistant. There is no clear understanding of why therapeutic response to corticosteroids in patients with PcP may vary in hosts with different causes of underlying immunosuppression. Provided that the efficacy of anti-*Pneumocystis* drugs in killing the fungus is independent of the patient condition, this host-to-host variation in response to treatment suggests the need to tailor anti-PcP therapy to the underlying cause of immunosuppression. A highly intriguing example is provided by patients with rheumatic diseases that may have different *Pneumocystis* burden thresholds for developing PcP (Shimada et al., 2018) suggesting that drugs used for underlying medical conditions could affect immune pathways differently and thus trigger different levels of immune responses (Shimada et al., 2018). In addition to PcP, corticosteroids are widely used clinically via the inhalation route to treat patients with asthma or chronic obstructive pulmonary disease (COPD) (Barnes, 2006; Adcock and Mumby, 2017) where steroid-resistance is observed (Christenson et al., 2019). Relatedly, pathways and signals activating mucus cell metaplasia in non-asthma-related chronic airway diseases like COPD and cystic fibrosis are induced by mediators that are distinct from those involved in allergic airway disease (McGuckin et al., 2015). All patients with COPD and some patients with asthma display steroid resistance that is to certain extent clinically unresponsive to corticosteroids (Barnes, 2013). Glucocorticoids exert their anti-inflammatory action by binding to the glucocorticoid receptor that inhibits transcription factors as c-Jun, NF-kB, and other signal transcription pathways that control the expression of pro-inflammatory mediators (Rhen and Cidlowski, 2005; Barnes, 2006). Some of these pathways are activated by *Pneumocystis* (Wang et al., 2005; Swain et al., 2012; Bello-Irizarry et al., 2014). They may also decrease mucin protein production by reducing transcription of mucin genes (Barnes, 2016). The wide anatomic availability of the glucocorticoid receptor explains that corticosteroids have multiple beneficial and detrimental effects (Rhen and Cidlowski, 2005; Barnes, 2006).

Results also suggest that the progressive increase in mucus, mCLCA3 and MUC5AC was also CLCA1-associated as demonstrated by mucus inhibition, lesser inflammation and improved survival after administration of the CLCA1 blocker NFA (Figures 8, 9). Induction of the CLCA1 pathway with increased mucins has been shown to correlate with *Pneumocystis* infection in humans (Vargas et al., 2013; Perez F. J. et al., 2014; Rojas et al., 2019) and in animal models (Swain et al., 2012; Eddens et al., 2016; Iturra et al., 2018; Rojas et al., 2019) and is strongly related to IL-13 driven mucus cell metaplasia, STAT6 activation and MUC5AC and MUC5B expression in the airways (Swain et al., 2012; Eddens et al., 2016; Iturra et al., 2018; Rojas et al., 2019). These models document increased IL-13 and mucus associated to *Pneumocystis* infection in the immunocompetent host providing an indication of activation of innate immune pathways. Germane to this work, it has been shown that NFA decreases mucus excess without affecting the expression of IL-13 receptors (Nakano et al., 2006) and, therefore, IL-13 was not measured in this model. Clinically, CLCA1 has been associated with innate airway immune response, airway inflammation (Long et al., 2006; Zhang and He, 2010), asthma (Hoshino et al., 2002; Mei et al., 2013), and COPD (Hauber et al., 2005; Hegab et al., 2007). CLCA1 may also act as a molecular signal to induce cytokine release by airway macrophages (Ching et al., 2013). All the above, suggests that CLCA1 protein is a relevant mediator of innate immune responses providing an explanation to the increase in survival of PcP-infected rats after administration of NFA via modulation of mucus responses. Furthermore, antibody blockage of the mouse homolog mCLCA3 suppresses symptoms and mucus overproduction in a murine model of asthma emphasizing the relevance of this pathway (Song et al., 2013).

Importantly, the exact contribution of mCLCA3 to mucus production is not completely elucidated. For example a knockout model to a putative transmembrane domain of mCLCA3 shows that MUC5AC production in response to an allergic challenge remained unchanged respect to wild type control mice (Robichaud et al., 2005) suggesting that mCLCA3 is not required for mucus production at least in its native conformation. Whether fragments of mCLCA3 could induce physiologic effects is unknown. This possibility is suggested by the demonstration that hCLCA1 and mCLCA3 are secreted non-integral membrane proteins (Gibson et al., 2005; Mundhenk et al., 2006). Beyond mCLCA3, many other pathways participate in mucus production. This is a complex process that involves transcription factors like STAT6, CREB, SP-1 and AP-1, the NAPDH oxidase component NOX-4, mitogen-activated protein (MAP) kinases and the transmembrane protein TM16A (Whitsett and Alenghat, 2015).

Niflumic acid is a non-steroidal anti-inflammatory drug and similar to glucocorticoids inhibits cyclooxygenase-2 (COX2) which is a potent mediator of inflammatory pathways. However, in this work NFA treatment led first to decrease mucus excess followed by decreasing inflammatory cuffing infiltrates evidencing a faster anti-mucus than anti-inflammatory effect. This suggests that the anti-mucus effect of NFA observed in our model would be unrelated to COX2 inhibition. Of note, administration of indomethacin, a non-selective inhibitor of cyclooxygenase 1 and 2, is not accompanied by mucus decrease (Nakano et al., 2006). Whether anti-inflammatory synergy between glucocorticoids and NFA occurs would be interesting to determine. This was not possible to evaluate in this model as steroids were given to all rats. Moreover, NFA is also a potent inhibitor of phospholipase A2 that favors hydrolysis of pulmonary surfactant (Cremonesi and Cavalieri, 2015). Hughes et al. (1998) documented that aerosolized administration of synthetic surfactant, which overcomes the activity of phospholipase A2, increases survival of rats with steroid-induced PcP to levels comparable to those obtained in this model (Figure 9). A potential effect of NFA in surfactant levels remains to be determined.

Data about NFA dosage, route of administration, tolerance and toxicity in rats is scarce despite previous use to inhibit goblet cell hyperplasia, airway hyperresponsiveness and mucus overproduction in murine models and in human bronchial epithelial cells. Therefore, we did preliminary experiments to document tolerance and safety of NFA in healthy rats. Results of these experiments indicate that for protective effects, NFA administration needs to be uninterrupted, using 7 days a week daily administration scheme (Figures 1B, 9). The survival advantage conferred by NFA was lost if continuous administration was not maintained (Supplementary Figure S3).

To show a mechanism of action for NFA effects in mucus production is beyond the reach of this work. However, regardless of the limitations of our model, this work demonstrates for the first time that NFA treatment effectively decreased inflammation, mucus production and significantly improved animal survival in steroid-induced PcP.

## Conclusion

Results advocate CLCA1-mediated innate immune responses having a probable causal relationship for *Pneumocystis* mediated mucus immunopathology in steroid-induced PcP and propose steroid resistant mucus as an explanation to the lack of response to steroids in patients with PcP. A fully specific target inhibition of CLCA1 will prove the concept. Drugs aiming to revert mucus excess during PcP may have a role as adjuvants to drugs with anti-*Pneumocystis* activity and the potential efficacy of therapies to block innate-immune pathways including CLCA1 protein needs to be explored in this condition.

In addition, taking together the potential of *Pneumocystis* to induce mucus-associated immunopathology and the striking anti-mucus effects documented for NFA, we recommend this steroid-induced PcP model as an appropriate tool to evaluate therapeutic approaches to control mucus excess in chronic respiratory diseases including steroid-resistant mucus excess.

## Data Availability

All datasets generated for this study are included in the manuscript and/or Supplementary Files.

## Author Contributions

FP and SV designed the study and wrote the manuscript. FP and CP performed animal experiments. PI and FP performed the morphometric determinations. FP performed the molecular determinations. FP, PI, FM, VG-A, and SV analyzed and interpreted data. All authors reviewed and contributed to the final version of the manuscript.

## Conflict of Interest Statement

The authors declare that the research was conducted in the absence of any commercial or financial relationships that could be construed as a potential conflict of interest.

## Supplementary Material

FIGURE S1 Safety of intraperitoneal administration of NFA in rats. NFA safety was tested at 3 or 12 mg/Kg/day doses administered via intraperitoneal injections during 28 consecutive days to healthy rats. Control animals received intraperitoneal injections with vehicle alone. **(A)** Animals treated with each NFA dose or with vehicle alone showed no significant differences in weight gain. **(B,C)** Safety blood tests showed all values within normal reference ranges for Sprague-Dawley rats. However, a dose-dependent increase in UREA levels was seen in NFA-treated animals, and a non- dose-dependent increase in ALKP in the NFA 3 mg/Kg/day-treated animals was also detected. NFA: Niflumic acid; CREA: Creatinine; UREA: Urea nitrogen; TBIL: Total bilirubin; DBIL: Direct bilirubin; AST: Aspartate transaminase; ALKP: Alkaline phosphatase; ALT: Alanine transaminase; GGT: Gamma glutamyl transferase. *n* = 5; ANOVA test: ^ns^no significant; ^∗∗∗^*P* < 0.001.

FIGURE S2 *Pneumocystis* spp.-DNA amplification in rats receiving steroids alone or steroids plus Niflumic acid. All rats received oxytetracycline starting 3 weeks before and throughout the duration of the experiment as depicted in Figure 1B.

FIGURE S3 Survival of rats after interruption of NFA administration. A 5-day a week administration scheme was attempted to reduce animal stress. NFA was delivered daily via intraperitoneal injection starting after 4 weeks of steroid administration (arrow) and interrupted after 5 days of NFA treatment (arrowhead and dotted line). *n* = 8; Mantel–Cox test: ^ns^ no significant.

DATA SHEET S1 Dataset for figures: MS Excel dataset tables for each figure.
