# Supplementary figures and images for: Niflumic Acid Reverses Airway Mucus Excess and Improves Survival in the Rat Model of Steroid-Induced Pneumocystis Pneumonia
Source: Front Microbiol. 2019 Jul 5;10:1522. doi: 10.3389/fmicb.2019.01522 (PMC6624676; doi:10.3389/fmicb.2019.01522)

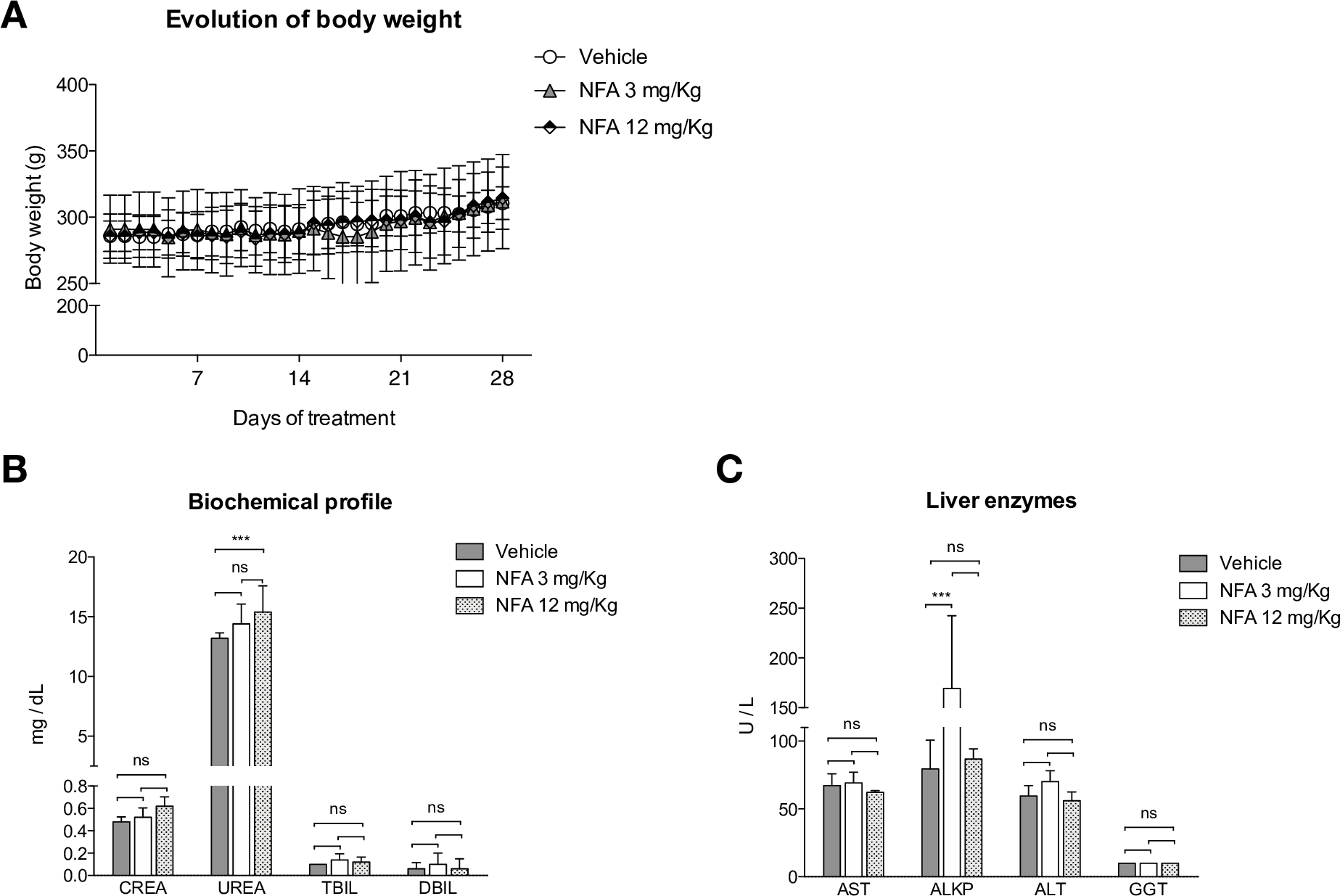

Supplement: FIGURE S1 — Safety of intraperitoneal administration of NFA in rats. NFA safety was tested at 3 or 12 mg/Kg/day doses administered via intraperitoneal injections during 28 consecutive days to healthy rats. Control animals received intraperitoneal injections with vehicle alone. (A) Animals treated with each NFA dose or with vehicle alone showed no significant differences in weight gain. (B,C) Safety blood tests showed all values within normal reference ranges for Sprague-Dawley rats. However, a dose-dependent increase in UREA levels was seen in NFA-treated animals, and a non- dose-dependent increase in ALKP in the NFA 3 mg/Kg/day-treated animals was also detected. NFA: Niflumic acid; CREA: Creatinine; UREA: Urea nitrogen; TBIL: Total bilirubin; DBIL: Direct bilirubin; AST: Aspartate transaminase; ALKP: Alkaline phosphatase; ALT: Alanine transaminase; GGT: Gamma glutamyl transferase. n = 5; ANOVA test: nsno significant; ∗∗∗P < 0.001. [file Image_1.TIF]

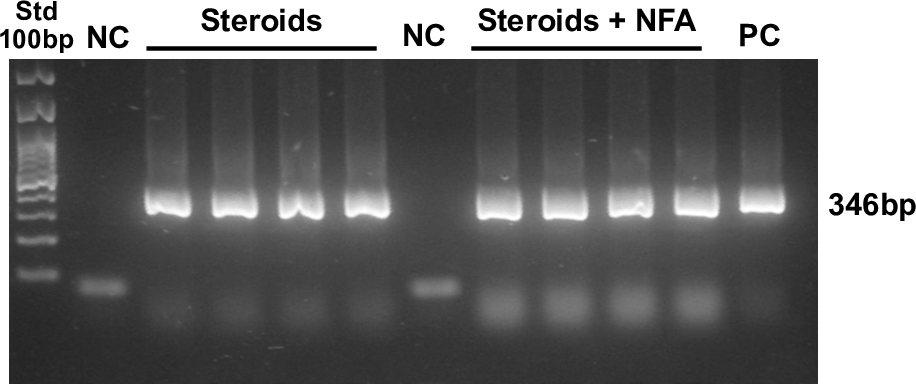

Supplement: FIGURE S2 — Pneumocystis spp.-DNA amplification in rats receiving steroids alone or steroids plus Niflumic acid. All rats received oxytetracycline starting 3 weeks before and throughout the duration of the experiment as depicted in Figure 1B. [file Image_2.TIF]

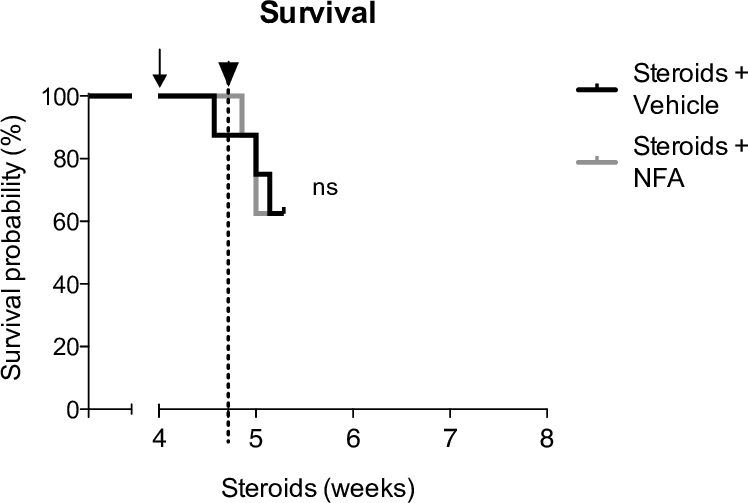

Supplement: FIGURE S3 — Survival of rats after interruption of NFA administration. A 5-day a week administration scheme was attempted to reduce animal stress. NFA was delivered daily via intraperitoneal injection starting after 4 weeks of steroid administration (arrow) and interrupted after 5 days of NFA treatment (arrowhead and dotted line). n = 8; Mantel–Cox test: ns no significant. [file Image_3.TIF]
